# Supplementary material for: Factors Associated with Undertaking Health-Promoting Activities by Older Women at High Risk of Metabolic Syndrome
Source: Int J Environ Res Public Health. 2022 Nov 30;19(23):15957. doi: 10.3390/ijerph192315957 (PMC9736211; doi:10.3390/ijerph192315957)
Supplement: Supplementary file 1 [file ijerph-19-15957-s001.zip › ijerph-1996158-supplementary.pdf]

Uczestniczy Pani/Pan w badaniach naukowych prowadzonych na Wydziale Fizjoterapii AWF Wrocław. Wypełniając ankietę zgadza się Pani/Pan na przetwarzanie swoich danych osobowych w celach związanych z realizacją projektu naukowego.

Imię i nazwisko: ……………………………………………. Nr tel.:…………………………………………………

Wiek: ……………… Wykształcenie:☐zawodowe ☐średnie ☐wyższe Zawód:…………….

Wzrost: ……… Masa ciała: ……… BMI: ………… Obwód talii: ……… Obwód bioder:………. WHR: ………..

1. Nadciśnienie………………………………………………….. ☐TAK ☐NIE ☐NIE WIEM Leczone: ☐TAK ☐NIE
2. Poziom cukru na czczo:………………………..…… Cukrzyca: ☐TAK ☐NIE ☐NIE WIEM Leczone: ☐TAK ☐NIE
3. Podwyższony poziom cholesterolu:…………………………... ☐TAK ☐NIE ☐NIE WIEM Leczone: ☐TAK ☐NIE
4. Podwyższony poziom trójglicerydów:…………………………☐TAK ☐NIE ☐NIE WIEM Leczone: ☐TAK ☐NIE
5. Czy pali Pani/Pan papierosy? ☐TAK ☐NIE
6. Dolegliwości bólowe ze strony kręgosłupa: ☐TAK ☐NIE
7. Dolegliwości bólowe innych stawów: ☐TAK ☐NIE Jakie?……………………………………………………….
8. Inne dolegliwości bólowe: ☐TAK ☐NIE Jakie?……………………………………………………….
9. Co jest Pani/Pana największym problemem zdrowotnym? ……………………………………………………………………..
10. Jakie choroby występują najczęściej w Pani/ Pana rodzinie? …………………………………………………………………...
11. Czy obawia się Pani/ Pan o swoje zdrowie? ☐TAK ☐NIE
12. Czy jest Pani/ Pan zadowolona/y ze swojej kondycji fizycznej/ sprawności? ☐TAK ☐NIE
13. Czy jest Pani/ Pan zadowolona/y ze swojej wagi? ☐TAK ☐NIE
14. Czy lekarz kiedykolwiek zachęcał Panią/ Pana do:

    zmiany trybu życia na aktywny? ☐TAK ☐NIE zmiany diety? ☐TAK ☐NIE zmniejszenia wagi ciała? ☐TAK ☐NIE
15. Jak obecnie spędza Pani/ Pan swój czas wolny? (proszę zaznaczyć maksymalnie 3 odpowiedzi)

    ☐ czytanie książek, oglądanie telewizji ☐ aktywność domowa (sprzątanie, gotowanie, opieka nad wnukami, praca w ogrodzie)

    ☐ spotkanie z rodziną, ze znajomymi ☐ sport i rekreacja fizyczna

    ☐ inne (jakie?)…………………………………………………………………………………………………..
16. Czy jest Pani/ Pan zadowolona/y z dostępu do usług rehabilitacyjnych finansowanych przez NFZ? ☐TAK ☐NIE
17. Czy obecnie korzysta Pani/ Pan z zajęć rekreacyjnych organizowanych dla osób starszych? ☐TAK ☐NIE

    Jakie to zajęcia?... Odpłatne? ☐TAK ☐NIE
18. Jakie są powody podejmowania przez Panią/ Pana aktywności fizycznej? (proszę zaznaczyć maksymalnie 3 odpowiedzi)

    ☐ poprawa/ utrzymanie kondycji fizycznej

    ☐ poprawa wyglądu/ zapobieganie otyłości

    ☐ poprawa stanu zdrowia

    ☐ zalecenia lekarza

    ☐ poprawa samopoczucia

    ☐ chęć przebywania z innymi ludźmi
19. Jakie są przeszkody w podejmowaniu przez Panią/ Pana aktywności fizycznej? (proszę zaznaczyć maksymalnie 3 odpowiedzi)

    ☐ brak czasu

    ☐ brak pieniędzy

    ☐ brak motywacji

    ☐ zły stan zdrowia

    ☐ brak takiej możliwości w pobliżu miejsca zamieszkania

    ☐ inne (jakie?)……………………………………………
20. Czy mając możliwość uczestnictwa w zajęciach ogólnousprawniających mających na celu poprawę sprawności fizycznej i stanu zdrowia, organizowanych w pobliżu miejsca zamieszkania skorzystałaby/ skorzystałby Pani/ Pan z nich? ☐TAK ☐NIE

……………………………………………………….

Podpis

You are participating in scientific research conducted at the Faculty of Physiotherapy, Wroclaw University of Health and Sport Sciences. By completing the questionnaire, you agree to the processing of your personal data for purposes related to the scientific project.

Name: .... Tel. no.:...

Age: .... Education: ☐ vocational ☐ secondary ☐ higher Occupation:...

Height:.................. Weight:.............. BMI:................ Waist circumference:............... Hip circumference:............... WHR: ....

1 Hypertension... ☐YES ☐NO ☐ I don't know Treated: ☐YES ☐NO

2. Fasting sugar level:.. Diabetes: ☐YES ☐NO ☐ I don't know Treated: ☐YES ☐NO

3. Elevated cholesterol:.. ☐YES ☐NO ☐ I don't know Treated: ☐YES ☐NO

4. Elevated triglyceride levels:... ☐YES ☐NO ☐ I don't know Treated: ☐YES ☐NO

5. Do you smoke cigarettes? ☐YES ☐NO

6. Complaints of back pain: ☐YES ☐NO

7. Complaints of pain in other joints: ☐YES ☐NO What?...

8. Other complaints of pain: ☐YES ☐NO What?...

9. What is your biggest health problem? ....

10. What diseases are most common in your family? ....

11. Are you concerned about your health? ☐YES ☐NO

12. Are you satisfied with your physical condition/fitness? ☐YES ☐NO

13. Are you satisfied with your weight? ☐YES ☐NO

14. Has your general practitioner ever encouraged you to:

change to an active lifestyle? ☐YES ☐NO change your diet? ☐YES ☐NO reduce your body weight? ☐YES ☐NO

15 How do you currently spend your free time? (please mark up to 3 answers)

☐ reading books, watching TV ☐ household activities (cleaning, cooking, taking care of grandchildren, gardening)

☐ meeting with family, with friends ☐ sport and physical recreation

☐ other (what?)..

16. Are you satisfied with access to rehabilitation services financed by the National Health Fund? ☐YES ☐NO

17. Are you currently taking advantage of recreational activities organized for the elderly? ☐YES ☐NO

What kind of activities are they?... Paid? ☐YES ☐NO

18 What are your reasons for engaging in physical activity? (please mark up to 3 answers)

☐ improving/maintaining physical fitness

☐ improving your appearance/ preventing obesity

☐ improve health

☐ prescribed by a doctor

☐ improving well-being

☐ willingness to spend time with other people

19 What are the obstacles to your undertaking physical activity? (please mark up to 3 answers)

☐ lack of time

☐ lack of money

☐ lack of motivation

☐ poor health

☐ lack of such opportunities near where you live

☐ other (what?)...

20. If you had the opportunity to participate in general fitness and health improvement activities organized near your place of residence, would you take advantage of them? ☐YES ☐NO

....

signature
